# How could an African swine fever outbreak evolve in an enzootic context? The case of Imerintsiatosika, Madagascar in 2015

**DOI:** 10.1371/journal.pone.0221928

**Published:** 2019-09-06

**Authors:** Harentsoaniaina Rasamoelina Andriamanivo, Dinaniaina Randriamananjara, Rijaniaina Ambinintsoa Ralalarison, Lalaina Arivony Nomenjanahary, Nivohanitra Perle Razafindraibe, Edithe Diana Andria-Mananjara, Domoina Oninirina Rakotomanana, Peter Samuel Fenozara, Marcellin Biarmann, Ariane Halm, Harimahefa Razafimandimby, Loïc Flachet, Eric Cardinale

**Affiliations:** 1 Epidemic Intelligence Unit, SEGA One Health Network, Indian Ocean Commission, Ébène, Mauritius; 2 Département Vétérinaire, Faculté de Médecine d’Antananarivo, Antananarivo, Madagascar; 3 Cabinet Vétérinaire, Imerintsiatosika, Arivonimamo, Madagascar; 4 Virology Unit, Institut Pasteur de Madagascar, Antananarivo, Madagascar; 5 Indian Ocean Field Epidemiology Training Programme, SEGA One Health, Network, Indian Ocean Commission, Ébène, Mauritius; 6 Direction des Services Vétérinaires, Antananarivo, Madagascar; 7 Département de Recherche Zootechnique, Vétérinaire et Piscicole), Antananarivo, Madagascar; 8 Centre International de Recherche Agronomique pour le Développement, La Réunion, France; The University of Melbourne, AUSTRALIA

## Abstract

African swine fever (ASF) is a haemorrhagic contagious pig disease generally causing high mortality. ASF is enzootic in Madagascar with outbreaks reported each year. An ASF outbreak occurred in May 2015 in the municipality of Imerintsiatosika in Madagascar. We investigated the outbreak to describe it and to identify risk factors in order to propose control measures, and to document evidence of an ASF outbreak in an enzootic country. We took biological samples from very sick and dying pigs, sold by the farmer to the butcher, for PCR analysis. An active search for all possible farm-cases was carried out. A definition of suspected farm-case was established and we implemented a descriptive survey and a retrospective cohort study. Laboratory results confirmed ASF virus infection. Suspected farm-cases represented 81 farms out of 922. Out of 3081 pigs of infected farms, 44% (95% CI: 42–46%) were sick, of which 47% were sold or slaughtered. Case fatality was 60% (95% CI: 56–63%) while 21% (95% CI: 19–24%) of the diseased pigs recovered. The outbreak duration was nine months and half of the infected farms’ pig population remained after the outbreak. Compared to the exotic breed, local pigs had twice the risk of infection. It is the first detailed report of an ASF outbreak in an enzootic situation. The disease still has a large impact with 50% animals lost. However, the case fatality is lower than expected that suggests the possibility of resistance and subclinical cases. Proximity to road and increased number of farms are risk factors so biosecurity measures are needed. Further studies are needed to understand why pigs of local breed are more affected. Finally, an acceptable alternative to the sale of sick animals should be found as this currently is the breeders’ means to reducing economic loss.

## Introduction

African swine fever (ASF) is a highly contagious haemorrhagic disease due to a virus of the Asfaviridae family, genus *Asfivirus* [1, 2]. It affects domestic and wild pigs. Soft ticks of the genus *Ornithodoros spp* play a vector and reservoir role [3, 4] but their presence is not always compulsory for the transmission cycle [5, 6]. All secretions and excretions from infected animals are infectious for several weeks, facilitating transmission by direct contact [5, 7]. The virus can also be transmitted via fomites such as contaminated clothes, shoes, equipment and vehicles, as it can persist in the environment for several days [5, 6]. In domestic pigs, ASF is a multisystem haemorrhagic disease with morbidity and mortality up to 100% [8, 9, 10]. All ages are equally affected [8, 9]. However, in enzootic countries, ASF is more frequently subclinical and chronic [5, 11, 12, 13, 14, 15]. Acquired resistance of pigs, lower virus virulence or higher genetic resistance of local breed pigs have been suggested as hypotheses to explain this situation [5, 16]. This resistance is not transmitted to offspring [17]. Despite this less pathogenic disease form, sporadic ASF outbreaks still occur in enzootic countries [13, 14, 18]. The disease constitutes a pig farming constraint and cause of important economic losses especially in developing countries in Africa [18], but also in eastern European countries [19, 20, 21]. and in Sardinia [22].

In Madagascar, ASF was introduced in late 1997 [23], and has since spread all over the country. The clinical picture was generally a haemorrhagic disease followed by death. Mortality reached up to 90% and caused a decrease of the national pig population by 60%, with a decline from 1.3 million to 0.5 million animals [23]. Since the middle of 1999, there were no more large epizootics and ASF has been considered enzootic with sporadic outbreaks [23]. To control the disease, policies were established, such as banning of free-range system, biosecurity requirements (fences, isolation), compulsory reporting and sanitary slaughtering [24]. However, eradication of the disease is difficult. ASF-infected *Ornithodoros moubata* soft ticks have been detected in the country [25, 26, 27, 28]. Conventional control methods like those mentioned are often impossible in countries where financial resources and veterinary services are limited [10, 29]. It has been reported that in case of ASF outbreaks, farmers in Madagascar rush to sell or slaughter their pigs to avoid economic losses [5, 30, 31], as do those in other African enzootic countries [9, 10, 32, 33].

Currently, ASF disease remains a major threat for farmers. Large commercial farms disappeared due to the threat of high economic losses and small-scale confined farms or free-range systems have been increasing instead [31]. The national pig population has increased and reached 1.5 million in 2014 [34], despite the presence of the disease. A study done in 2006 at the slaughterhouse of Arivonimamo district, one locality of high pig concentration, showed that one quarter of apparently healthy slaughtered pigs had antibodies against ASF [35]. This result suggest that subclinical cases are possible.

The ongoing animal diseases surveillance system was set up with help of the Indian Ocean Commission as part of the “Surveillance Epidémiologique et Gestion des Alertes” (SEGA) One Health regional network [36]. It was based on 23 sentinel veterinarians located in the different agro-ecological areas of Madagascar. This sentinel surveillance includes 23 out of the 114 districts of Madagascar [36]. ASF is among the priority diseases targeted by the surveillance system. From July to October 2015, in those sentinel districts, this surveillance detected 36 Fokontany (lowest administrative unit composed of neighbouring villages) affected by swine fever [36], generally with only one or two concerned farms each. It was a clinical surveillance to detect any suspected case. Therefore, it was always difficult to distinguish between classical and African swine fever since both circulate in the territory. Nevertheless, the low number of infected farms reported by field veterinarians, at national level, raised question on a possible decrease of ASF contagiousness and/or greater resistance of pigs against the virus. Investigating ASF outbreak was necessary in order to assess the current burden of disease, the evolution of ASF outbreaks in terms of duration, mortality, contagiousness and of risk factors associated to disease spread or to higher resistance of pigs.

In late April 2015, the sentinel surveillance system detected a suspected swine fever alert originating from the field veterinarian of Imerintsiatosika, 28km from the capital Antananarivo. As part of the SEGA One Health network, together with the veterinary services of Madagascar, we conducted an outbreak investigation. Our objectives were to:

- Assess incidence, mortality, case fatality and duration of the outbreak.
- Quantify the farmers behaviours in terms of emergency sale or slaughtering.
- Identify risk factors for virus spread and pigs’ case fatality.

## Materials and methods

### The SEGA one health network

This study was done under the framework of SEGA One Health network (www.reseausega-coi.org/en) [37]. It is an intergovernmental network that operates on epidemiological surveillance and response to outbreaks in the five countries of the Indian Ocean Commission (Madagascar, Mauritius, Comoros, Seychelles, France/Reunion Island). Interventions in health monitoring and in outbreak investigation, with the national authorities, both for human and for animal health, in those member States is one of the mandates of the SEGA One Health network according to its charter.

The coordination team of the SEGA One Health network, responded to a request from the veterinary services of Madagascar to support them in conducting this outbreak investigation.

### Study site and pig farming

**Fig 1:**
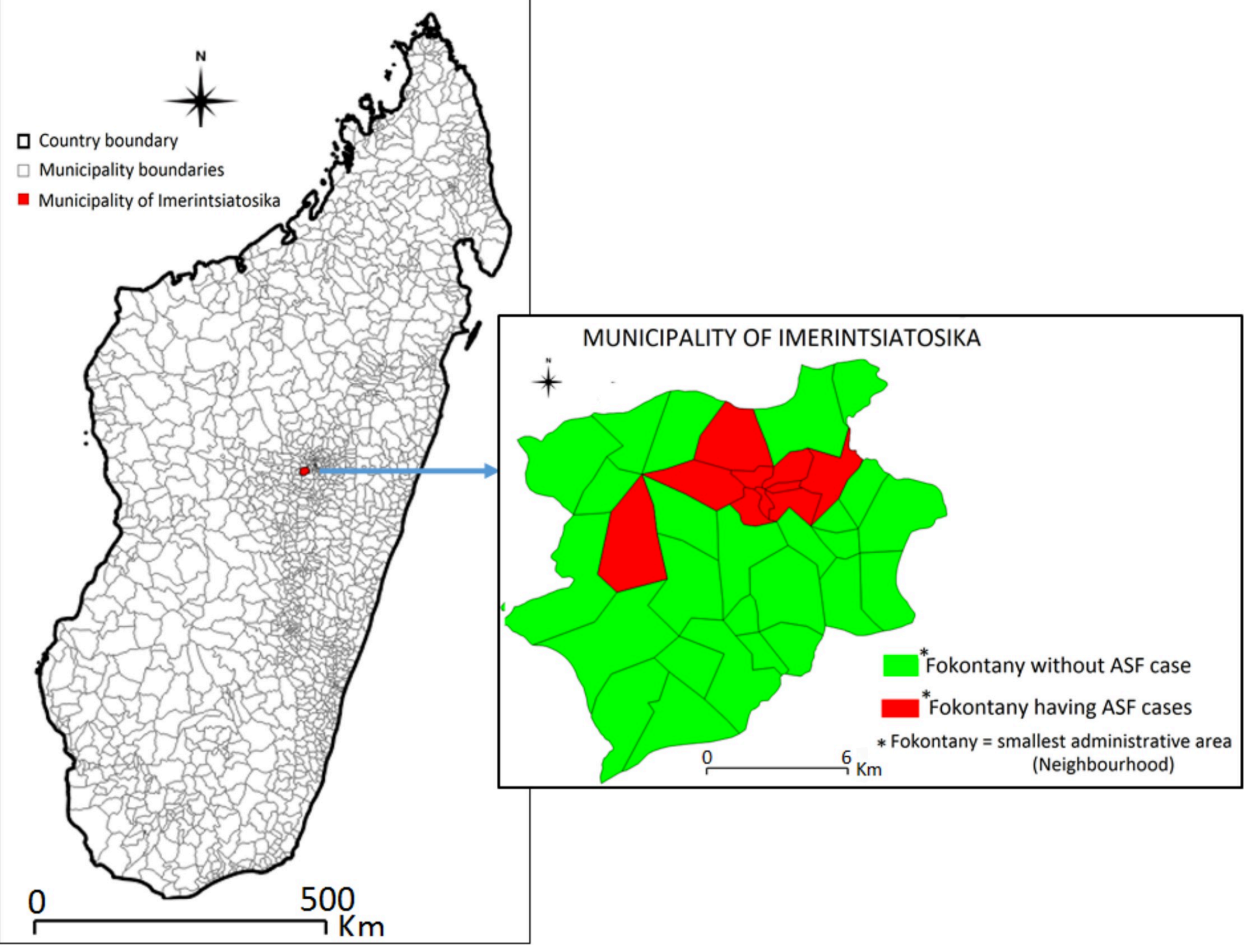
Location of the municipality of Imerintsiatosika and the areas affected by ASF.

The study site was the municipality of Imerintsiatosika (Fig 1). Its surface is 173 Km^2^ and it is divided into 36 Fokontany. The population density is 310 people/Km^2^ that makes it one of the most populated municipalities in the country. The national road that leads to the Mid-West region of Madagascar passes through the municipality.

In terms of pig farming, there were an estimated of 2000 farms and 7000 pigs in Imerintsiatosika. Out of the 1693 municipalities nationwide, it has the highest pig population of both, local and exotic phenotype breeds. It is one of the main supplier of the capital city with pork. Imerintsiatosika has also the highest number of commercial farms, which range from simple enclosures to well-built brick-fired houses with cement floors. However, it is still a context of small breeding system in a developing country [31]. Within each Fokontany, houses are generally gathered in one location and the rest of the area is mainly for agriculture. In this situation, there are many “farms” in a very small area. It is easy to see two or more farms beside each other, separated only by some meters. Furthermore, type of farms and husbandry practices are at a very low level of biosecurity, even those fenced. Fences are only 2 or three pieces of wood. There is no particular restriction and no biosecurity measures taken when there is visitor coming into the farm. There are also movements of animals for natural reproduction because the breeder brings the female to another farm having a male.

This area is under the supervision of one field veterinarian who has a staff of ten animal health workers.

## Confirmation of diagnosis

The first step was to confirm that it was an ASF outbreak. It is a common practice in pig husbandry in Madagascar to slaughter pigs in case of swine fever. Slaughtering means that farmers decided to slaughter themselves for their own consumption, or to call for a butcher. In the field, there is not a system of culling and compensation from the government. To avoid a total loss, the breeders are used to calling butchers who slaughter, buy the animals and sell the meat. The investigation team waited for this kind of opportunity to get samples. When a breeder called for a butcher for a very sick or recently deceased pig, the field team came also and ask the consent of breeders, to take spleen. Conventional PCR analysis was done at the “Institut Pasteur de Madagascar” (IPM) following the protocol described by Randriamparany [38].

### Case definitions

We established case definitions for possible and suspected farm-cases.

A possible farm-case was a farm identified as affected by the ASF outbreak by key informants (veterinarian, his staff and Fokontany chief).

We defined a suspected farm-case as a possible farm-case where, from September 2014 to June 2015, at least two of the following clinical signs were reported from its pig population:

- Digestive signs: diarrhoea or vomiting or constipation
- External haemorrhagic signs: skin redness or petechia or epistaxis
- Respiratory signs: dyspnea or cough or nasal discharge
- Nervous signs: ataxia or paralysis or muscle tremor
- Death
- General sign: listlessness
- Haemorrhagic lesion of spleen or kidney or mediastinal lymph node

### Data collection

We performed an outbreak investigation during May 2015 with both a descriptive survey and an analytical study. Follow-up of the outbreak continued till end of June. All data collection was done by the same team that was trained before the field activities.

For the descriptive survey, we did a census of all the farms of the municipality. We performed an active search for farm-cases, Fokontany by Fokontany. We surveyed farm-cases with help of a standardized questionnaire and after verbal consent of each farmer. For each farm, we collected data about the beginning (first sick animal) and the end (last dead or recovered animal) of the disease within it, the total number of pigs stratified by breed and age group. The breed could be local or improved (all exotic breeds, pure or crossed). The age groups were piglet, grower and adult, the latter classified into breeding pigs, female or male, in activity or in pre-culling. For each breed and age group, we collected data on sick and dead pigs. We also recorded the number of pigs for each possible outcome at the moment of the survey: healthy, dead, sold or slaughtered with precision if they were symptomatic or not. Finally, we gathered all clinical signs reported by the farmer during the disease circulation in his farm.

Concerning the analytical study, we performed a retrospective cohort study to identify risk factors for morbidity. The unit we used was the individual pig. We considered the entire pig population from suspected farm-cases as a cohort. We collected data on the pigs’ disease status (sick or not). We also recorded age group and breed as exposure variables.

### Data analysis

For the descriptive survey, we assessed possible farm-cases which matched the suspected farm-case definition and kept them for data analysis. We checked the spatial distribution of suspected farm-cases by calculating the proportion of affected farms per Fokontany. Thereafter, we mapped the spread of the disease over the different Fokontany. We mapped also the cumulative incidence by Fokontany for the whole period of the outbreak and we compared it with the population density and the density of farms per Fokontany. We compared proportions of affected Fokontany close to the national road *versus* away from the road, then those having human population density above 400 people/Km vs those below this value, and finally those having above 35 pig farms/Km2 *vs* below this value. Chi-squared test or Fisher exact test was used for this purpose.

To assess evolution over time, we built the epidemic curve using the starting date of the disease in the farm-cases. Most of farmers could only remember if the starting date was on early, middle or near the end of the month. Then, we used ten day periods as time units. We also calculated the disease duration and its quartiles for all suspected farm-cases. Regarding the clinical presentation of ASF, we calculated the proportions of suspected farm-cases that exhibited each clinical sign in at least one pig.

At individual level (pig), we calculated the incidence (number of sick pigs out of the total of pigs during the outbreak). Then, we calculated the frequencies of emergency sale and slaughtering of sick or healthy pigs. We continued with case-fatality (number of dead pigs out of the sick ones). For these indicators, we calculated overall estimates and then estimates by breed and by age group.

In the retrospective cohort study, we compared the incidence by breed and then we calculated crude relative risks (RR) and their 95% confidence intervals (CI_95%_). Then, we stratified by age-group. We tested for the existence of interaction between age-group and breed with the Breslow-Day test and by verifying potential overlaps between CI_95%_ of stratum-specific RR.

## Results

The field team could collect two spleen in an infected farm and the diagnostic of ASF was confirmed. This confirmation allowed us to move forward into the description and analyse of the outbreak.

### Outbreak description

Of 36 Fokontany in the municipality, 10 (28%; 95% CI: 14–43%) were affected by the ASF outbreak. These affected Fokontany included 922 pig farms of which 116 (12%; 95% CI: 10–14%) were identified as possible ASF farm-cases. Out of 116 possible farm-cases, 86 (74%) accepted to participate in the study. Of these, 81 farms fulfilling the suspected farm-case definition were included in the data analysis. Herd-sizes of suspected farm-cases ranged from 1 to 265 with a median of 15 pigs. All of them were small-scale fenced farms.

**Fig 2:**
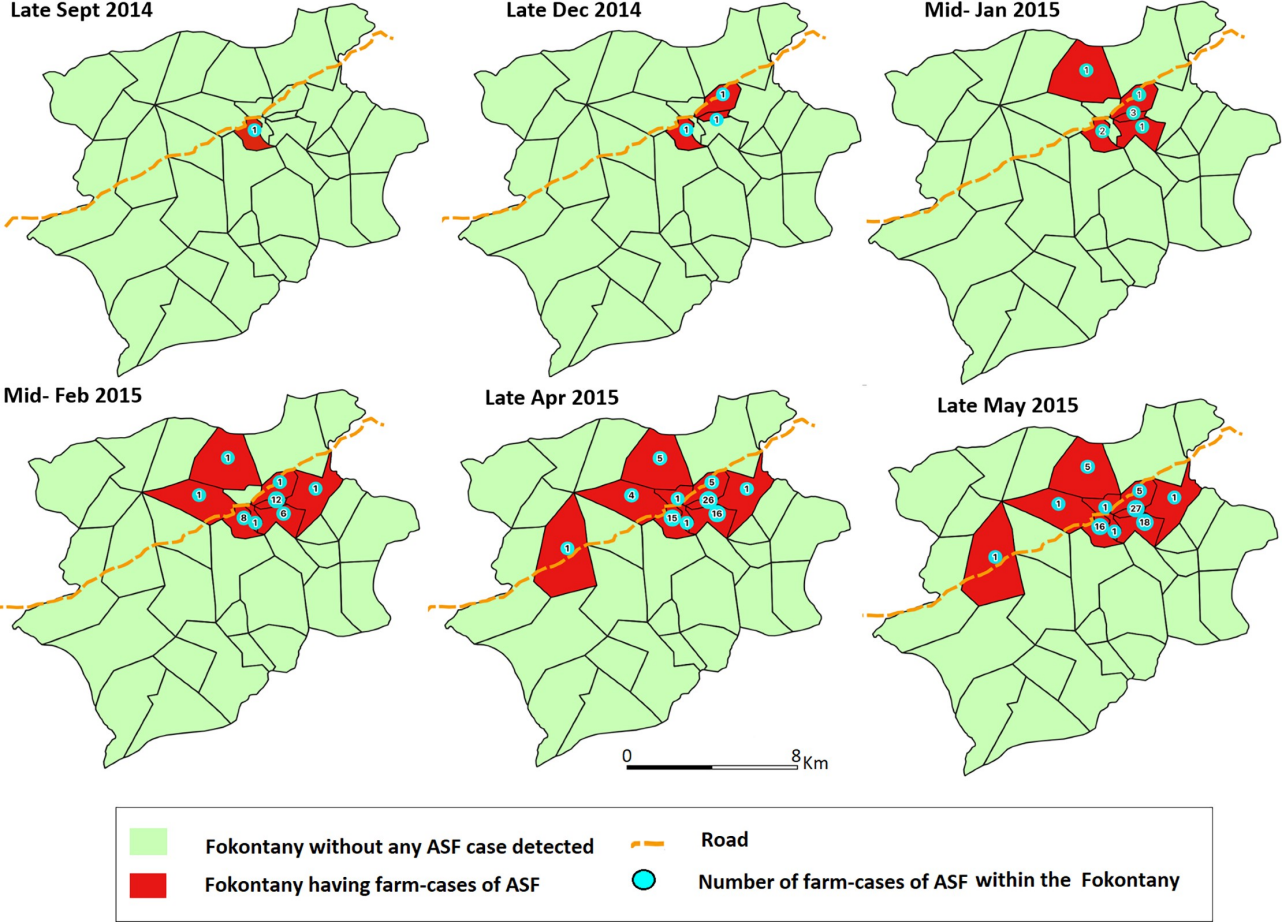
Outbreak of ASF in Imerintsiatosika Madagascar in 2014–2015: Spread over the Fokontany.

The outbreak started in a farm located in a Fokontany called Labrousse in late September 2014. In December 2014, it reached two additional Fokontany and spread at a rhythm of two new Fokontany each month till the ten Fokontany were affected in late April 2015 (Fig 2).

**Fig 3:**
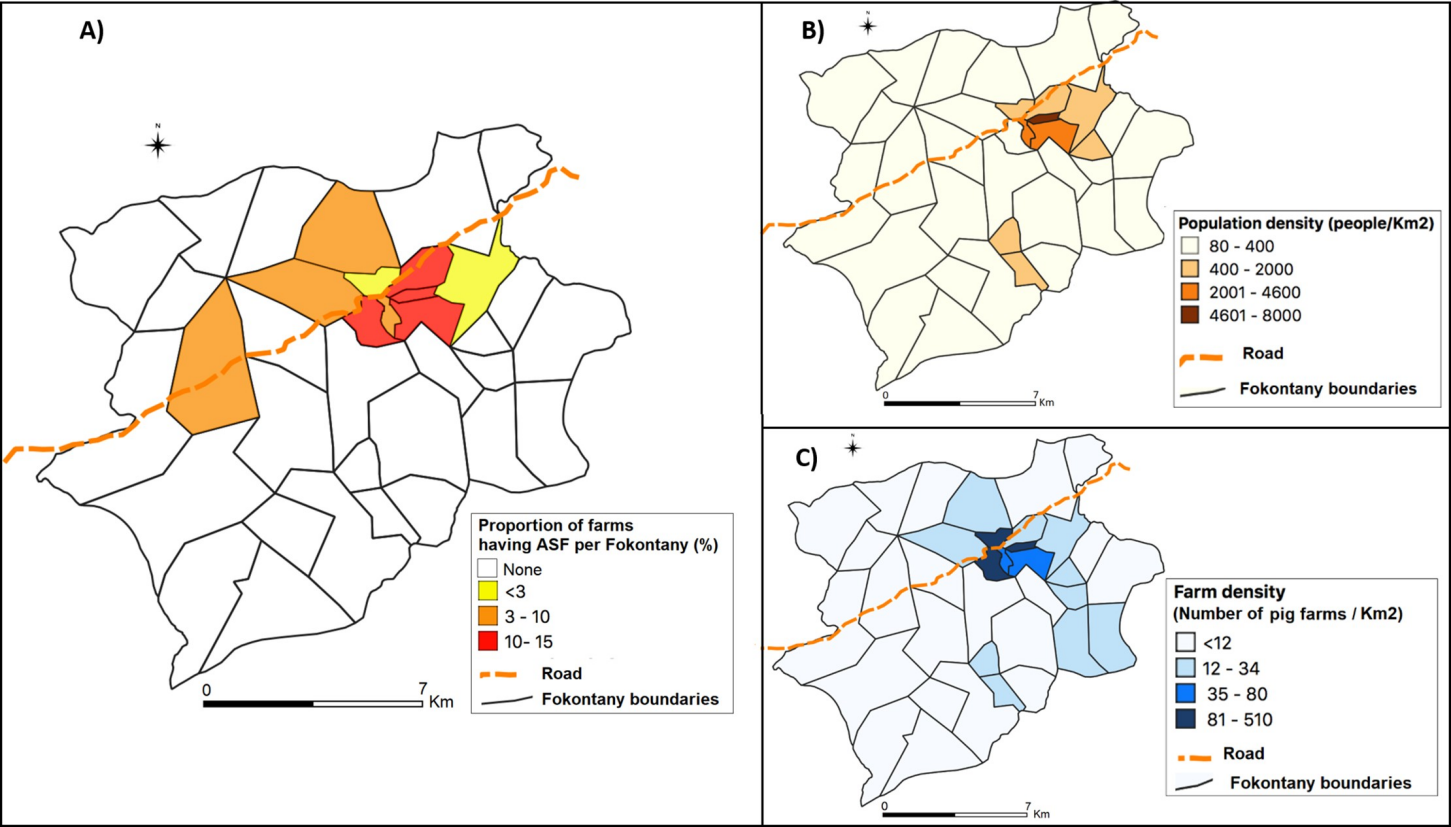
Outbreak of ASF in Imerintsiatosika Madagascar in 2014–2015: Cumulative incidence of suspected farm-cases (A), population density (B) and density of pig farms per Fokontany (C).

Proportions of suspected farm-cases by Fokontany ranged from 2% to 15% (Fig 3A). Out of 10 Fokontany next to the national road, 7 were affected compared to 3 out of those away from the road (p = 0,001). Six out of 9 Fokontany with a population density above 400 people/Km^2^ them were affected by this outbreak of ASF (Fig 3B), compared to 4 out of 23 having a population density below this value (p = 0.006). Five Fokontany had above 35 pig farms/Km2 and all of them were hit by the outbreak (Fig 3C).

**Fig 4:**
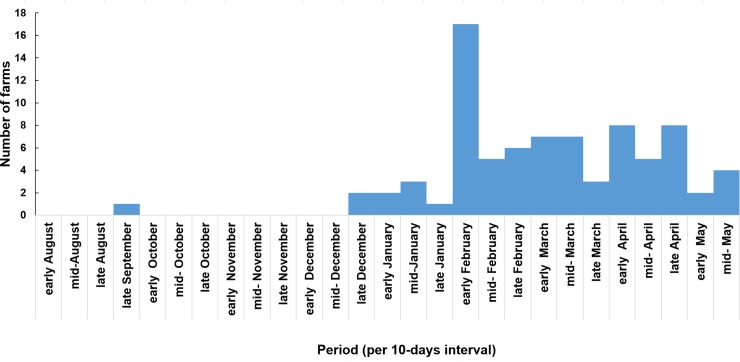
Suspected farm-cases of ASF by date of first within-farm disease onset, in 2014–2015 in Imerintsiatosika, Madagascar (n = 81).

The outbreak began in late September 2014 and ended in late May 2015. The highest peak was observed in early February 2015, followed by two lower peaks in April (Fig 4). Within-farm disease duration ranged from 1 to 90 days, with a median of 11 days. Quarter of farms reported disease outbreaks lasting 21 days or more.

The clinical signs reported by the 81 suspected farm-cases were: digestive signs (62 farms, 77%; 95% CI: 67–86%), external haemorrhagic signs (57 farms, 70%; 95% CI: 60–80%), nervous signs (53 farms, 65%; 95% CI: 55–76%), death (51 farms, 63%; 95% CI: 52–73%), listlessness (47 farms, 58%; 95% CI: 47–69%), respiratory signs (44 farms, 54%; 95% CI: 43–65%) and haemorrhage of internal organs (25 farms, 31%; 95% CI: 21–41%). Among the digestive signs, constipation was recorded for 40 farms (49%; 95% CI: 38–60%).

The pig population of the 81 suspected farm-cases was 3081 heads. Table 1 shows the pigs’ outcomes following the outbreak. The overall disease incidence, from September 2014 to May 2015, was 44% (95% CI: 42–46%), ranging from 4% to 100% within farm-cases. Case fatality among diseased pigs that were not removed from the farms was 60% (95% CI: 56–63%). Twenty-one percent (21%; 95% CI: 19–24%) of diseased pigs recovered.

**Table 1:** Outcome of pigs from suspected farm-cases following the ASF outbreak, Imerintsiatosika, Madagascar 2015.

Age-group	Breed	Pig population	Total diseased		Diseased and lost to follow-up			Sold healthy		Dead		Recovered	
			Number	Incidence (%)	Sold sick	Slaughtered	% of total diseased	Number	% of pig population	Number	Case fatality*(%)	Number	% out of total diseased
Piglets	Local	14	9	64	9	0	100	0	0	0	0	0	0
	Exotic	800	403	50	58	24	20	117	15	205	64	116	29
Growers	Local	149	140	94	47	6	38	0	0	17	20	70	50
	Exotic	1064	344	32	137	20	46	342	32	129	65	58	21
Adults	Local	386	265	69	210	0	79	0	0	54	98	1	0
	Exotic	668	199	30	116	7	62	57	9	30	39	46	23
	TOTAL	3081	1360	44	577	57	47	516	17	435	60	291	21

* Case fatality = Number of dead / (Total of diseased—Diseased and lost of sight) X 100

In terms of farmers’ behaviour, 35% (95% CI: 34–37%) of pigs were sold urgently, of which 577 (53%; 95% CI: 50–56%) were ill. Farmers slaughtered 57 (4%; 95% CI: 3–5%) of the diseased pigs. A total of 634 diseased pigs were sold and/or slaughtered and therefore lost to follow-up.

The pig population that remained in affected farms after the outbreak was of 1496 animals, 49% (95% CI: 47–50%) of the initial number.

### Risk factors

The retrospective cohort analysis revealed that 75% of 549 local breed and 37% of 2532 exotic breed pigs were ill during this ASF outbreak; the crude RR was 1.99 (CI_95%_: 1.86–2.13). Regarding the incidence by age group, compared to 44% of 1054 adults, 51% of 814 piglets (RR_crude_ = 1.15; CI_95%_: 1.04–1.27) and 41% of 1213 growing pigs (RR_crude_ = 0.93; CI_95%_: 0.86–1.02) were affected.

Table 2 shows the RRs taking into account pig breed and age-group. There was an interaction between these two factors with incidence a. The CI_95%_ of the adjusted RR of the different strata did not overlap and the Breslow-Day test was highly significant with p = <10^−3^ for the incidence, but not applicable to the case fatality. In terms of incidence, the risk for local breed pigs to be affected by the disease was 2.9 times and 2.3 times higher for growing pigs and for adults respectively, compared to those of the exotic breed. For piglets, the risk of ASF was the same for both breeds.

**Table 2:** Adjusted relative risks from pigs’ breed stratified by age-group, retrospective cohort during ASF outbreak, Imerintsiatosika Madagascar 2015.

Age-group	Breed	Total	Diseased	Incidence (%)	RR	95% CI	p
Piglets	Local	14	9	64	1.28	(0.86–1.90)	0.30
	Exotic	800	403	50	Ref		
Growers	Local	149	140	94	2.91	(2.64–3.20)	0.00
	Exotic	1064	344	32	Ref		
Adults	Local	386	265	69	2.30	(2.01–2.64)	0.00
	Exotic	668	199	30	Ref		
	TOTAL	3081	1360	44			

## Discussion

This was the first time an ASF outbreak in a small town of a rural area in Madagascar was studied in detail using descriptive and analytical methods. We describe the evolution of an ASF outbreak in an enzootic area, which might reflect the situation in other enzootic countries. Our findings describe its evolution over time and over the Fokontany that composed the municipality. We describe also the impact of the outbreak in terms of incidence, case-fatality at animal level and the pigs’ outcomes following the outbreak, including farmers behaviour in the face of the outbreak. This investigation also allowed the identification of risk factors associated with incidence at animal level.

The disease concerned one third of all Fokontany within the municipality of Imerintsiatosika and around one tenth of the farms of those Fokontany. Forty-four per cent of the pig population from affected farms were sick. Case fatality was 60% in the diseased pigs still accountable for at investigation time (not sold and not slaughtered) but for sure it was underestimated. It is likely that a proportion of the diseased pigs that were sold or slaughtered would have succumbed to ASF. Furthermore, the breeders generally are used to selling the animals in worst conditions and the butcher is the main customer.

Compared to the situation following ASF introduction in Madagascar in late 1997, with a large-scale epizootic and mortality of up to 100% [23], the impact of the disease in our study was lower. These findings coincide with cases described in other ASF enzootic countries, such as Malawi, Mozambique and Nigeria, where mortality was around 50–60% during recurrent outbreaks [11, 14, 15, 16, 18, 39, 40, 41, 42]. However, with one third of Fokontany and more than 80 farms affected, and only half of the pig population remaining at the end of the outbreak, the disease was still devastating for affected farms in Imerintsiatosika.

The suspected origin of the outbreak was an introduction of local breed pigs from north-west Madagascar. In fact, veterinary services recorded a probable ASF outbreak that was not laboratory-confirmed in the north-western part of the country during the last third of 2014. There is an important and regular supply, generally monthly, of local breed pigs transported from this area to several farms in Imerintsiatosika [43, 44]. Introducing pigs from a free-range system is a well-known risk factor for introducing ASF virus in fenced farms [18].

The epidemic curve shows one main peak followed by waves. The outbreak started in September 2014 and the peak was observed only in early February 2015. The disease spread silently between some farms and Fokontany before the main peak. The rhythm was of 2 new Fokontany infected per month. The waves of the epidemic curve probably corresponded to secondary farm-to-farm spread via direct contact or fomites. Factors such as the crossing national road, bigger population density and pig farms density are possibly related to a spread of the virus among farms via fomites. Actually, the results show higher frequencies of Fokontany affected when they were close to the road, or had more inhabitants and pig farms within. Those factors could be interpreted as proxy indicators for more circulation and contact between farms. But further study would be necessary to confirm and quantify the role of those factors in terms of increasing risk of ASF in farms. However, as high pig farm density was reported to be a risk factor of ASF elsewhere [9, 38, 45], as was proximity of a main road [45] we recommend that farms should increase their biosecurity measures.

The outbreak in Imerintsiatosika lasted nine months. The disease remained over three weeks in 25% of affected farms and 21% of diseased pigs recovered. Despite Imerintsiatosika has higher pig population and pig farms compared to other municipalities, it is still a context of small breeding system with very low level of biosecurity, even those fenced. The disease should have spread quickly within farms. A similar situation was observed elsewhere in enzootic countries [46, 47]. In Gulu district in northern Uganda, the duration of ASF disease per village varied between a couple of weeks to 7 months, and many survivors were recorded [46, 47]. Our study results suggest a lower impact of ASF in some pigs [26, 36]. A previous study in the district of Arivonimamo, of which Imerintsiatosika municipality is part, showed a seroprevalence of 24% in asymptomatic pigs slaughtered at the slaughterhouse [36]. The presence of antibodies in healthy pigs was described in enzootic countries such as Senegal [12], Uganda [8] and Nigeria [13, 14]. This seroprevalence suggests that the virus has been circulating in this area and that subclinical cases can occur. Acquired resistance, induced through previous exposure to lower doses of virus, or with viruses of reduced virulence, has been documented [5, 16]. Ticks of the genus *Ornithodoros* are present in Madagascar [25, 26, 27]. A previous study confirmed that these soft ticks were infected by ASF virus [25]. Their role in the enzootic status of ASF in Madagascar was then suggested [28], and might further explain the presence of resistant pigs here, as in other enzootic countries [40, 41]. Resistance might be expressed through subclinical or chronic disease that could lead to “carrier animals” shedding virus for several weeks and thus supporting the enzootic ASF circulation [46, 48]. Those resistant pigs could also explain the increased disease duration within 24% of affected farms. Such farms became a virus source over long time periods [46, 48] and might have constituted a source of infection for secondary farms, explaining the overall duration of outbreak. Further studies should be carried out to explore the mechanisms of this potential resistance to ASF.

A factor that could have limited the spread of virus towards more Fokontany and farms was the type of farming system. The outbreak started in the centre of Imerintsiatosika, where there are no free-ranging pigs. Our results confirmed that all suspected farm-cases were semi-scale fenced farms. Compared to a free-range farming system, a known risk factor for rapid spread of the virus [10, 16, 49], such a confined housing system slows down the virus spread.

Looking at farmers’ behaviours, one third of pigs from affected farms were sold, but only 4% were slaughtered. Most sold pigs would eventually also have been slaughtered locally or in the capital, after passing through a complex network of middlemen. Previous studies [23, 30] have already mentioned these behaviours. These findings confirmed that emergency sale or slaughtering are one of the main mitigation strategies that farmers adopt in case of diseases of economic importance [9, 32, 33]. These practices, common in African enzootic countries [9, 32, 33], especially for small-scale farmers [18], are known to favour virus spread [50]. Even if they are forbidden according to the current regulation against ASF [24], pig husbandry is important for these small-scale farmers’ livelihoods [18, 42], and not selling pigs would mean a total loss because there is no government compensation. Further efforts are required to find the appropriate balance between measures that are acceptable for farmers and regulations’ requirement [48; 50]. Participatory epidemiology and cost-benefit analysis would be suitable tools for tackling this [50]. Some strategies that have shown effectiveness in mitigating ASF impact elsewhere should be considered, such as: food and water control, isolation/separation of sick pigs, washing/disinfection of farm equipment and tools [39]. It would be more feasible for small-scale farms to implement such measures than for those practising free-range systems [45].

In this outbreak the local breed had a higher risk of infection. This could be explained by those small-scale farmers sourcing their new pigs of local breed mainly from free-range areas [31, 43, 44], which are known as systems at risk of ASF [9, 10]. Actually, for growing and for fattening in fenced farms, many breeders are used to buying local piglets or growers of local breed from remote villages practising free-range system. Sometimes, they mix those local breed pigs with exotic breed pigs sourced from elsewhere. The called “exotic breed pigs” in the country includes all pigs having the exotic phenotype even if they are crossed breed. However, as soon as the virus is introduced in a farm, all the pigs, whatever their breed, are exposed. Further studies are needed to understand why local breed had higher risk on infection.

Our study had some limitations:

- First, we had 21% of non-response among the 116 possible farm-cases. The reasons were that farmers were afraid to be accused of introducing ASF in the locality or of possible control measures such as culling. There is a chance that the situation in these farms could have changed the investigation results. However, following our discussions with Fokontany chiefs, we believe that non-response farms had the same behaviour as included farms.
- Secondly, in accordance with ethical consideration, only two pigs were sampled for laboratory confirmation and ASF diagnosis. Moreover, our definition of a suspected farm-case could have coincided with other diseases, mainly with classical swine fever (CSF). Therefore, we cannot completely exclude possible false positives among the suspected farm-cases. However, even if theoretically possible, simultaneous ASF and CSF outbreaks are not common [30].
- Thirdly, we might under-estimate the disease burden in terms of incidence. The apparently healthy pigs that were sold could have been under incubation period and developed the disease later. Otherwise, the last suspected farm-case that we detected was in mid-May. We continued the investigation up to one month after mid-May we did not find any other case in the area of study. However, we cannot exclude that the outbreak continued after our investigation.
- A possible bias was recall bias, as collected information referred to past events for most of the farmers. However, pig production is among the main income sources in the municipality and herd sizes are not big. This leads us to assume that events related to this outbreak, which caused important economic loss, would be well remembered. However, it would have been difficult to remember the exact date of onset of the disease or the exact clinical sign showed by each animal. We considered it in the protocol and that was the reason why the epidemic curve was by a 10 days period instead of weekly or daily, and why the case definition was at farm-level instead of individual animal. It was easier for the farmer to remember if the disease started in the early, mid or late of the month.

In conclusion, Imerintsiatosika is a small town with a high density of small-scale pig farms. ASF has become enzootic in Madagascar, leading to a lower incidence, a lower case fatality and a longer duration of outbreak. Nevertheless, the disease burden in affected farms was high, and only half of the pig population remained after the outbreak. High risk behaviour like emergency sale was very common to mitigate the farmers’ economic loss. To increase effectiveness of control measures, regulations should be reviewed and carefully consider barriers and incentives for farmers [48], instead of the conventional approach [10, 18]. Local breed pigs were more at risk of acquiring ASF. The virus possibly spread from farm to farm with fomites playing an important role. Farmers should enhance their biosecurity measures concerning visitors.

## Supporting information

S1 Table Data. (XLSX)
